# Mini-Review: The Administration of Apoptotic Cells for Treating Rheumatoid Arthritis: Current Knowledge and Clinical Perspectives

**DOI:** 10.3389/fimmu.2021.630170

**Published:** 2021-02-25

**Authors:** Eric Toussirot, Francis Bonnefoy, Charline Vauchy, Sylvain Perruche, Philippe Saas

**Affiliations:** ^1^INSERM CIC-1431, Centre d'Investigation Clinique Biothérapie, Pôle Recherche, CHU de Besançon, Besançon, France; ^2^Fédération Hospitalo-Universitaire INCREASE, CHU de Besançon, Besançon, France; ^3^Rhumatologie, Pôle PACTE (Pathologies Aiguës Chroniques Transplantation Éducation), CHU de Besançon, Besançon, France; ^4^Département Universitaire de Thérapeutique, Université de Bourgogne Franche-Comté, Besançon, France; ^5^Univ. Bourgogne Franche-Comté, INSERM, EFS BFC, UMR1098, Interactions Hôte-Greffon-Tumeur/Ingénierie Cellulaire et Génique, LabEx LipSTIC, Besançon, France; ^6^MED'INN'Pharma, Besançon, France

**Keywords:** rheumatoid arthritis, cell-based therapy, apoptotic cells, efferocytosis, inflammation

## Abstract

Rheumatoid arthritis (RA) is a chronic immune-mediated disease managed by conventional synthetic drugs, such as methotrexate (MTX), and targeted drugs including biological agents. Cell-based therapeutic approaches are currently developed in RA, mainly mesenchymal stroma cell-based approaches. Early-stage apoptotic cells possess direct and indirect anti-inflammatory properties. During the elimination of dying cells (a process called efferocytosis), specific mechanisms operate to control immune responses. There are compelling evidences in experimental models of arthritis indicating that apoptotic cell administration may benefit joint inflammation, and may even have therapeutic effects on arthritis. Additionally, it has been demonstrated that apoptotic cells could be administered with standard treatments of RA, such as MTX or TNF inhibitors (TNFi), given even a synergistic response with TNFi. Interestingly, apoptotic cell infusion has been successfully experienced to prevent acute graft-vs.-host disease after hematopoietic cell transplantation in patients with hematologic malignancies, with a good safety profile. In this mini-review, the apoptotic cell-based therapy development in arthritis is discussed, as well as its transfer in the short-term to an innovative treatment for patients with RA. The use of apoptotic cell-derived factors, including secretome or phosphatidylserine-containing liposomes, in RA are also discussed.

## Introduction

Rheumatoid arthritis (RA) is a chronic immune-mediated disease that primarily affects the peripheral joints. This inflammatory rheumatic disease is responsible for joint inflammation, cartilage, and bone destruction that progressively lead to joint deformations, structural damage, and disability (1). Rheumatoid arthritis is associated with specific comorbidities, such as an enhanced cardiovascular risk (2), but also systemic complications and premature death (1). Dramatic improvements have been done these past 20 years in the diagnosis and management of RA. Indeed, new classification criteria have been proposed, and new treatment paradigms together with the development of novel therapeutics have considerably improved the prognosis of the disease (3, 4). Currently, the treatment of RA is based on conventional synthetic drugs [conventional synthetic disease modifying antirheumatic drugs (csDMARDs)] or targeted drugs, including targeted synthetic and biological drugs (tsDMARDs and bDMARDs, respectively) (5). The treatment strategy of RA has been now clarified, and specific international recommendations help the physician in the drug choice and the therapeutic management, taking into account disease activity and severity (6). However, a substantial proportion of patients does not respond adequately to these drugs and, some of them do not achieve remission or low disease activity (7), the therapeutic goals in RA. In addition, the long-term use of these drugs could lead to severe adverse events (8).

Cell-based therapies have been developed in the treatment of RA (9). Administration of cells that can modulate or even dampen the inflammatory process of RA is an exciting therapeutic challenge. Preliminary data with the administration of hematopoietic or mesenchymal stem cells in phase 1/2 clinical trials are currently available in patients with RA (9). In these approaches, the mode of action of hematopoietic or mesenchymal stem cells is still under debate. Administration of apoptotic cells could represent another relevant cell-based therapy for the treatment of RA (10). This cell therapy—developed in different experimental indications, such as transplantation or immune-mediated diseases—has been shown to modulate the immune response and to control, at least partially, the inflammatory process in experimental models of arthritis (10, 11). Thus, in this review, we will comment the current knowledge of apoptotic cell-based treatment in arthritis, and discuss the potential use of this cellular therapy in patients with RA.

## Rheumatoid Arthritis: A Brief Overview of Its Pathophysiology

The exact cause of RA remains currently unknown. Its development results from the complex interplay between genetic, environmental, hormonal, and psychological factors (12). Rheumatoid arthritis is characterized by inflammation and hyperplasia of the synovium, leading to the so-called rheumatoid “pannus.” Joint inflammation in RA is related to an immune activation with the participation of both innate immune cells [i.e., neutrophils, monocytes and macrophages, NK cells, dendritic cells (DC), mast cells, and innate lymphoid cells], and adaptive immune cells (Th1 and Th17 cells, B cells, and plasma cells). A strong cellular response has been initiated within the synovium with the activation of local synovial cells, including fibroblast- and macrophage-like synoviocytes (13). These resident synovial cells exhibit an aggressive phenotype with the production, together with immune cells, of a complex network of soluble factors, i.e., cytokines and chemokines, perpetuating the cellular activation and inflammatory reaction (14). Resident synoviocytes also produce metalloproteases that degrade the extra-cellular matrix components, i.e., cartilage and subchondral bone, ultimately promoting joint destruction (15). Rheumatoid arthritis is characterized by the production of well-described autoantibodies, of which rheumatoid factors (RF) and anti-citrullinated protein antibodies (ACPA) are the most common (16). ACPA can bind citrullinated motifs expressed by several self-proteins, including vimentin, fibrinogen, fibronectin, α-enolase, or type II collagen. The presence of RF and/or ACPA is associated with a more severe disease. Post-translational protein modifications explain the reaction of citrullination under the influence of the peptidyl-arginine deiminase enzymatic activity, with subsequent immunization and the production of ACPA (17). Other post-translational reactions have been described in RA, such as the formation of carbamylated residues promoting the synthesis of anti-carbamylated antibodies (18).

The treatment of RA is now well-defined, and has profoundly changed over the past 20 years (4). Methotrexate (MTX) is used as first line csDMARDs, and is considered as the reference treatment in RA (19). Targeted drugs (such as tsDMARDs or bDMARDs) are proposed as second line therapy in the case of inadequate response to csDMARDs (20). Among bDMARDs, TNFi have been available on the market for more than 20 years, and are widely used in clinical practice. Other bDMARDs include IL-6 blocking agents, a T cell costimulation blocker (abatacept) and a B cell depleting agent (rituximab) (21). Janus kinase inhibitors (tofacitinib, baricitinib, upadacitinib and filgotinib) correspond to tsDMARDs. These agents interfere with signal transduction involved in T cell activation (22).

## Cell-Based Therapies in Rheumatoid Arthritis

Cell-based therapeutic approaches have been recently developed in RA. The rationale is based on the administration of cells exhibiting direct or indirect anti-inflammatory properties targeting inflammatory cells involved in the RA pathophysiology (9). The cells used in RA are mainly stem cells from the hematopoietic or the mesenchymal lineage. Hematopoietic stem cells (HSC) possess the property of multilineage differentiation, resulting in all blood cell types. They are isolated from bone marrow, peripheral blood after G-CSF mobilization, or umbilical cord. The curative effect of hematopoietic cell transplantation (HCT) is based on the conditioning regimen that eliminates pathogenic immune cells and the re-establishment of the host immune system. This procedure has been performed in a limited number of RA patients [reviewed in (9)]. In this setting, allogeneic HCT is tolerated and can improve disease activity and/or maintain remission for the short term. However, these patients received a cytotoxic regimen before transplantation with potential immunologic or infectious complications, and are at risk of developing severe acute graft-vs.-host disease (GvHD). Another approach is the administration of mesenchymal stem/stroma cells (MSC). These cells are multipotent stromal cells that can differentiate into different cellular lineages. They derive from bone marrow, adipose tissue, umbilical cord, or other organs including the synovium. They exhibit several immunomodulatory effects, including: inhibition of T cell activation/proliferation, suppression of B cell activation and antibody production, reduction of NK cell activity, generation of regulatory T (Treg) cells, and polarization of macrophages toward an anti-inflammatory phenotype (9). In a large cohort of patients who had inadequate response to csDMARDs, MSCs were administrated intravenously together with csDMARDs and compared to patients who received only csDMARDs. A significant improvement of RA was observed in the MSC group, with a remission sustained up to 3–6 months (23). Another phase 1/2 clinical trial evaluated the safety and efficacy of allogeneic expanded adipose tissue-derived MSCs administered intravenously. That dose escalation, placebo controlled study consisted of 3 infusions over 2 weeks. The treatment was well-tolerated and yielded clinical improvement after 1 month. However, the efficacy was not sustained and the clinical response was mitigated at month 3 (24). Other cellular therapies have been tried in patients with RA, including the intra-articular (IA) administration of autologous tolerogenic DC, leading to promising results (25).

## The Immunomodulary Properties of Apoptotic Cells

Apoptosis is a physiological cell death process that occurs during the development, and throughout the life in all tissues. Every day, a billion of cells die and are eliminated without deleterious consequences as inflammation or inadequate immune response (26). Apoptosis is followed by the efficient clearance of dead cells by professional phagocytes, a mechanism called efferocytosis. Efferocytosis is a key mechanism for tissue homeostasis (26). The appropriate efferocytosis of apoptotic neutrophils is a major event in the resolution of inflammation (27). Conversely, a defective efferocytosis may contribute to impaired tissue homeostasis, delayed tissue repair, and non-resolving inflammation. In this context, inadequate clearance of apoptotic cells may promote auto-inflammatory and/or autoimmune diseases (28). Altered efferocytosis has been reported in RA (28, 29).

**Figure 1:**
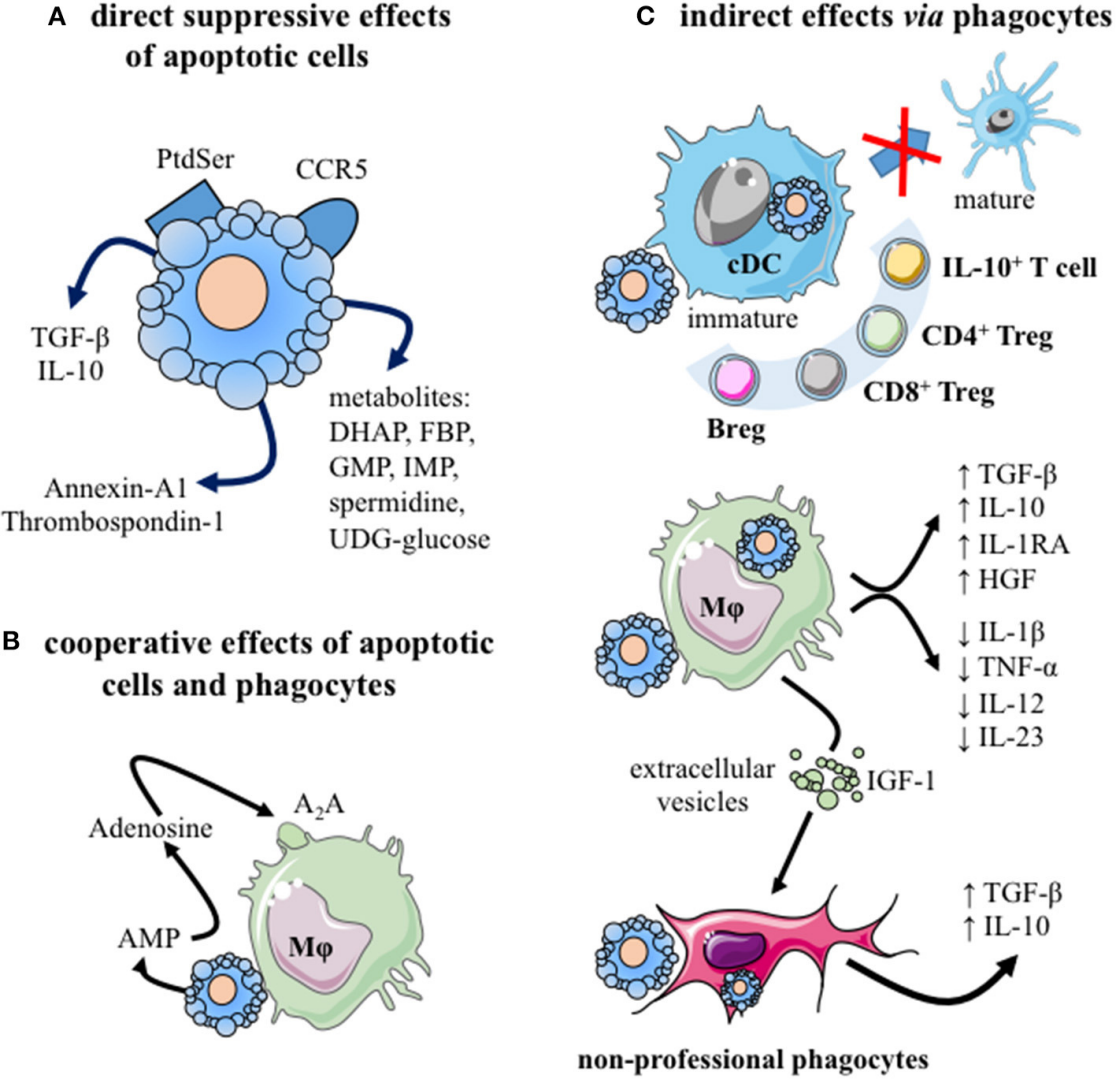
The immunomodulatory effects of apoptotic cells. **(A)** Apoptotic cells exert direct anti-inflammatory effects by: (i) neutralizing inflammatory chemokines, via CCR5 expression (30), and (ii) releasing different factors, including: anti-inflammatory cytokines [e.g., TGF-β (31)], mediators [i.e., annexin-A1 (32) or thrombospondin-1 (33)], as well as cellular metabolites [e.g., spermidine, guanosine monophosphate, inosine monophosphate (34)]. **(B)** Cooperation between apoptotic cells and their phagocytes may mediate immunosuppression. Apoptotic cells release adenosine monophosphate (AMP) which is then converted into adenosine by phagocyting macrophages. This factor interacts with macrophage adenosine A_2A_ receptors and stimulates anti-inflammatory gene expression (35). **(C)** Different phagocytes propagate the immunomodulatory effects of apoptotic cells after efferocytosis. Professional phagocytes (immature cDC and macrophages), as well as amateur phagocytes (e.g., fibroblasts, epithelial cells) may participate to efferocytosis (10). The consequences for phagocytes differ depending on the considered phagocytes. Efferocytosis by cDC blocks them in an immature stage or induces a tolerogenic profile that generates regulatory adaptive immune cells: IL-10-secreting cells, CD4^+^ Treg, CD8^+^ Treg, and/or IL-10-producing Breg cells. After efferocytosis, macrophages secrete anti-inflammatory factors (e.g., TGF-β, IL-10, IL-1RA, and HGF) and reduce their production of inflammatory cytokines (e.g., TNF-α, IL-1β, IL-12, and IL-23). Efferocytosis triggers macrophage reprogramming toward a pro-resolving profile (26, 27). Moreover, macrophages may stimulate via extracellular vesicles factors and IGF-1 the efferocytic capacity of amateur phagocytes (36). Finally, these amateur phagocytes may secrete immunosuppressive cytokines, such as TGF-β or IL-10. A_2_A, Adenosine 2A receptor; AMP, adenosine-monophosphate; Breg, regulatory B cells; cDC, conventional dendritic cell; DHAP, dihydroxyacetone phosphate; FBP, fructose-1,6-biphosphate; GMP, guanosine-5′ -monophosphate; HGF, hepatocyte growth factor; IGF-1, insulin-like growth factor; IL-, interleukin; IMP, Inosine-5′-monophosphate; Mϕ, macrophage; Ptdser, phosphatidylserine; TGF-β, transforming growth factor-beta; Treg, regulatory T cells. This figure was depicted, in part, by using Servier Medical Art, https://smart.servier.com/.

Apoptotic cells display immunomodulatory properties that can be harnessed for therapeutic use (10, 11). Indeed, both apoptotic cells themselves and the interaction of apoptotic cells with professional phagocytes are associated with immune regulation mechanisms (Figures 1A–C). Before being removed, apoptotic cells create a local transient suppressive microenvironment (30–34) (Figure 1A). In response to specific signals released by apoptotic cells, called “find me” signals, professional phagocytes move toward the sites where cells are dying (37–39). Professional phagocytes correspond mainly to tissue-resident and monocyte-derived macrophages, as well as specific DC subsets (10). Apoptotic cell engulfment is then driven by specific signals expressed on apoptotic cells or fragments of apoptotic cells [collectively called “don't eat me” and “eat me” (i.e., phosphatidylserine; Ptdser) signals] (40). While “don't eat me” signals prevent the uptake of viable cells, “eat me” signals stimulates the engulfment and modulate the immune responses. Thus, liposomes expressing Ptdser have been shown to mimic some immunomodulatory effects of apoptotic cells (41). Apoptotic cells may cooperate with macrophages to induce an immunosuppressive environment (Figure 1B) (35). After efferocytosis, an anti-inflammatory reprogramming of macrophages is initiated (26, 27), leading to a sustained immunosuppressive environment (Figure 1C) through the release of several anti-inflammatory cytokines [e.g., TGF-β, IL-10 (42, 43)], and other regulatory factors (44, 45), such as pro-resolving mediators (10, 11, 46). Reprogramming of macrophages also includes a decreased capacity to produce pro-inflammatory cytokines (e.g., TNF-α, IL-1β, IL-12, or IL-23) (10). Phagocyting dendritic cells are also maintained in an immature stage and/or developed a tolerogenic profile, contributing to the generation of regulatory immune cells, including IL-10-producing T cells, CD4^+^ Treg, CD8^+^ Treg or regulatory B (Breg) cells (Figure 1C). This tolerogenic microenvironment participates in the inhibition of Th1 and Th17 responses (10, 11). Overall, the administration of apoptotic cells may control an ongoing pro-inflammatory process by different regulatory mechanisms and by inhibiting pathogenic cells. All these mechanisms support the rationale to use apoptotic cell administration in chronic systemic inflammation, like in arthritis (46). We thus reviewed the effects of apoptotic cells, apoptotic cell-derived factors, approaches mimicking apoptotic cell injection, or factors released during efferocytosis in animal models of arthritis. A systematic literature search was performed in PUBMED Medline database until November 2020, using the following keywords: [apoptotic cells] OR [apoptotic cell factors] OR [efferocytosis factors] AND [arthritis] AND [animal models]. After title and abstracts selection, duplicate elimination and manual search, 10 articles were finally selected for analysis (34, 47–55) (Table 1).

**Table 1:** Experimental studies evaluating the effects of apoptotic cell administration or apoptotic cell-derived factors in animal models of arthritis.

**Reference**	**Experimental model of arthritis**	**Apoptotic cell source or apoptotic cell mimetic**	**Route of administration**	**Main effects**	**Final result and duration of the effect**
(47)	Collagen-induced arthritis (DBA/1 mice)	Syngeneic early apoptotic thymocytes	IV or IP	Reduction of collagen auto-antibodies; induction of IL-10-producing CD4^+^ T cells; reduction of IFN-γ secreting CD4^+^ T cells; IL-10-producing marginal zone B cells	Prophylactic effect on arthritis
(51)	Collagen-induced arthritis (DBA/1 mice)	Syngeneic early apoptotic thymocytes	IV	Reduction of collagen auto-antibodies; induction of collagen-specific Treg cells; resistance of APC to TLR ligand activation, indicating APC reprogramming; effects dependent on TGF-β; synergic results with TNFi	Transient therapeutic effect on arthritis
(55)	Collagen-induced arthritis (DBA/1 mice)	Secretome of macrophages eliminating apoptotic cells	IV or IP	Generation of collagen-specific Treg cells; pro-tolerogenic reprogramming of pDC and macrophages; mediated at least in part by TGF-β	Therapeutic effect on arthritis up to 60 days following secretome administration
(49)	Methylated BSA-induced arthritis (C57BL/6 mice)	Syngeneic early apoptotic thymocytes	IV	Suppression of Th17 response in the draining lymph nodes; increase of IL-10-producing T cells; IL-10-producing marginal zone B cells; effects dependent on natural IgM	Prophylactic effect on arthritis
(50)	Methylated BSA-induced arthritis (C57BL/6 mice)	Syngeneic apoptotic dendritic cells	IV	The use of activated apoptotic cells induces IL-6 and prevents TGF-β mediated prevention of arthritis	Prophylactic effect on arthritis
(47)	Serum transfer-induced arthritis (C57BL/6 mice)	Syngeneic early apoptotic thymocytes	IV or IP	No prophylactic effect	No improvement of arthritis
(34)	Serum transfer-induced arthritis (C57BL/6 mice)	Apoptotic cell-derived metabolites^*^	IP	Diminution of paw swelling	Prophylactic effect on arthritis
(52)	Lyme arthritis (C3H/HeJ mice infected with *Borrelia burgdorferi*)	Xenogeneic Jurkat leukemic cells	IA	A trend toward higher IL-10 levels in the joints	Therapeutic effect on arthritis (accelerated disease resolution)
(53)	Adjuvant arthritis (Lewis rat)	Phosphatidylserine-containing liposomes	IM	Reduction of *RANK* and *RANKL* mRNA; inhibitory effect on osteoclast formation; increased production of TGF-β and PGE2	Therapeutic effect on arthritis (reduction of paw volume at day 28, then rat sacrifice)
(54)	Adjuvant arthritis (Lewis rat)	Phosphatidylserine-containing liposomes	IM	Reduction of *RANK* and *RANKL* mRNA; Increased IL-10 and decreased IL-1β and IL-17 in the ankle joint; shift from IL-1β- to IL-10-producing infiltrated macrophages	Therapeutic effect on arthritis (reduction of paw volume at day 28, then rat sacrifice)
(48)	Streptococcal wall-induced arthritis (Lewis rat)	Xenogeneic early apoptotic thymocytes	IP	Decrease in the pro-inflammatory response of peritoneal macrophages; induction of Treg cells in the draining lymph node; effects dependent on TGF-β	Prophylactic effect on arthritis

*APC, antigen presenting cells; BSA, bovine serum albumin; IA, intra-articular ankle injection; IM, intramuscular (in the hind limbs); IP, intraperitoneal; IV, intravenous; pDC, plasmacytoid dendritic cell; PGE2, prostaglandin-E2; TNFi, TNF inhibitors; Treg, CD4^+^ FoxP^+^ regulatory T cells*.

* *Two cocktails were tested: one with six metabolites [dihydroxyacetone phosphate (DHAP), fructose-1,6-biphosphate (FBP), guanosine-5′-monophosphate (GMP), Inosine-5′-monophosphate (IMP), spermidine, UDG-glucose] and the other with 3 metabolites (GMP, IMP, and spermidine)*.

## The Effects of Apoptotic Cells in Preclinical Model of Arthritis

Six articles were selected (47–52). Apoptotic cell-based therapies have been evaluated through intravenous (IV), intraperitoneal (IP), or IA administration of early stage apoptotic cells in the main arthritis models. The main results are summarized in Table 1. According to the experimental model used and to the time of apoptotic cell injection (i.e., at the time of arthritis induction, before clinical symptom occurrence, or during ongoing arthritis), one may distinguish preventive vs. curative effects. A preventive effect of apoptotic cell administration on clinical scores has been observed in all the arthritis models tested (47–50), except in the serum transfer-induced arthritis (STIA) model (47). This negative result may be explained by the experimental model used, lacking the adaptive immune response (46). Indeed, implication of IL-10-producing T cells [T regulatory 1 (Tr1) cells], Treg and/or IL-10-secreting Breg cells has been shown to be triggered by apoptotic cell infusion in arthritis models and associated with the reduction of joint inflammation (47–49) (Table 1). In streptococcal cell wall (SCW)-induced arthritis, IP injection of apoptotic cells along with arthritis induction induced a significant improvement of joint inflammation and limited joint destruction (48). In this rat model, the results are more impressive on the chronic stage of the disease (48). A therapeutic effect of IV apoptotic cell administration has been reported in ongoing arthritis using the collagen-induced arthritis (CIA) model (51). In this model, co-administration of MTX with apoptotic cells induces arthritis reduction that was similar to apoptotic cell injection alone, thus demonstrating that MTX does not interfere with apoptotic cell infusion (51). The concomitant administration of a TNFi together with apoptotic cells induces better arthritis reduction (51), indicating a synergistic effect between the mode of action of both therapies. Finally, IA apoptotic cell injection has exerted also a therapeutic effect in a mouse Lyme arthritis model (52). Altogether, these different experimental results well illustrate the beneficial consequences of apoptotic cell administration in acute and chronic arthritis, with both preventive and curative effects. Apoptotic cell-based therapy may thus be potentially able to control ongoing clinical arthritis, to dampen inflammation and attenuate destructive articular changes. This strongly supports the therapeutic use of apoptotic cells in patients with RA.

## The Effects of Apoptotic Cell-Derived Factors in Preclinical Model of Arthritis

Four articles were selected for this review (34, 53–55) (Table 1). It has been shown that the intramuscular (IM) administration of Ptdser-containing liposomes in adjuvant arthritis (AA) is able to inhibit AA-induced trabecular bone loss (53). The same group demonstrated that this approach induced macrophage reprogramming with the reduction of IL-1β release and an increase in IL-10 production (54). This confirms that Ptdser-containing liposomes can mimic some effects of apoptotic cells (41). Recently, the administration of a mixture of 3 or 6 metabolites identified in apoptotic cell secretome has been shown to diminish paw swelling in the STIA model (34) (Table 1). These apoptotic cell-derived metabolites are able to control macrophage reprogramming toward a pro-resolving profile (34). Thus, selective apoptotic cell-derived factors may also be considered as innovative therapeutic approaches in arthritis. Furthermore, the secretome of macrophages eliminating apoptotic leukocytes has been shown to exert a therapeutic effect on arthritic clinical score in CIA by inducing collagen-specific CD4^+^ Treg cells and reprogramming plasmacytoid dendritic cells (pDC) and macrophages toward a tolerogenic state (55). This treatment of arthritis has been associated with a restored functional resolution of inflammation (47). This confirms the main role of efferocytosis in the control of inflammation (27), as well as the importance of the macrophage/pDC/Treg cell crosstalk in apoptotic cell-induced tolerance (56). Overall, apoptotic cell “derivatives” can be an alternative to apoptotic cell-based therapy.

## Apoptotic Cells in the Treatment of Arthritis: Toward A Clinical Use

Results from experimental studies detailed above support the rational for a clinical perspective in RA. This is based on the preclinical data, but also on an extensive research in the fields of cancer and transplantation (10, 11, 46). However, there are several concerns before translating these results to a clinical trial in humans. First, in most of the experimental models used to evaluate the apoptotic cell-based approach, arthritis is induced by a known and specific auto-antigen, which is not the case in patients. Second, certain models are self-resolving, without a chronic phase after the acute peak of the disease. This does not mimic the clinical situation. Third, most of the data have been obtained with a therapy given at the initiation of the disease, even before the onset of arthritic clinical symptoms. This is not feasible in clinic. Despite the fact that a mouse is not human and that regulatory mechanisms may differ between these two species (57), the feasibility must be discussed. What type of cell has to be used as apoptotic cells? Indeed, peripheral blood leukocytes are the easiest cell source to consider in human, contrary to apoptotic cells used in experimental models that are from the thymus, the spleen, DCs, and even leukemic cells (Table 1). As observed in experimental settings, apoptotic leukocytes would need to be at an early apoptotic stage, i.e., annexin-V^+^, 7-aminoactinomycin D or propidium iodide negative. These cells may be collected using cytapheresis. Different procedures may render the cells apoptotic and the different stimuli include γ-, X-ray, or UV-irradiation, Fas-mediated cell death, as well as corticosteroid treatment. The appropriate route of administration for the patients is the IV route, since this approach seems appropriate for a systemic disease, such as RA. This allows a quick diffusion of apoptotic cells through the body. To summarize, one may propose the following procedure for the apoptotic cell preparation in a clinical trial: a cytapheresis collecting peripheral blood leukocytes that are then exposed to a 35-Gy dose of γ- or X-ray irradiation to induce apoptosis. In these irradiation settings, one may obtain around 40–50% of cells in an early apoptotic stage, with a limited number of cells (<10%) in a necrotic stage. The concomitant use of csDMARDs (such as MTX) and of bDMARDs (TNFi) is not a limit, since MTX does not alter the therapeutic effects of apoptotic cells (51), whereas TNFi displays a synergistic effect (51). Glucocorticoids (GCs) are still used in clinical practice for the treatment of RA, and this medication may be administered with apoptotic cells. Indeed, GCs enhance apoptotic cell removal during the different phases of apoptotic cell clearance (40). The safety of apoptotic cell infusion is another concern. This cell-based therapy has been previously used in a clinical trial in the setting of HCT (58). In this phase 1/2a trial, donor early apoptotic cells have been administered intravenously the day before allogeneic HCT in order to prevent acute GvHD in 13 patients with hematological malignancies. The administered cells have been obtained from hematopoietic cell donors after cytapheresis. This cell-based therapy has been well-tolerated without related serious adverse events. The overall incidence of stage II to IV GvHD was 25% while in the group of patients who received the highest dose of apoptotic cells, this rate was 0% (58). The conclusion of this trial was that apoptotic cell infusion during HCT is a safe procedure and potentially effective in the prevention of GvHD. This illustrates the feasibility of this cell therapy approach. Considering these results, we plan to start a clinical trial on apoptotic cell infusion in RA patients who do not respond adequately to at least one bDMARD. The APO-RA project is a phase 1/2 trial that proposes one infusion of peripheral blood leukocytes rendered apoptotic cells by X-ray irradiation (ClinicalTrials.gov identifier: NCT02903212).

## Conclusion and Perspectives

The use of dying cell or their byproducts as a therapeutic approach in the treatment of RA, or other forms of arthritis, still represents an exciting and innovative therapeutic opportunity. Besides cell-targeted therapies that are currently used in RA (e.g., abatacept or rituximab), and besides MSC or tolerogenic DC administration, apoptotic cells represent another cellular approach with an attractive mode of action. There are compelling evidences demonstrating that apoptotic cell administration may dampen the inflammatory process of RA. Preliminary data in the setting of HCT indicate the feasibility of this approach with a good safety profile. It is, thus, now time to translate all these preclinical data in clinical settings. Some questions persist such as the exact therapeutic protocol to use (number of cells to administer, stimulus to generate early apoptotic cells without high a rate of necrotic cells), and whether a single infusion of apoptotic cells will give a sustained response.

## Author Contributions

ET performed the bibliographic search, wrote the first version of the manuscript, and made the table. PS completed the bibliographic search, edited the first version of the manuscript, and made the figure. All authors reviewed and edited the manuscript.

## Conflict of Interest

FB and SP are employees by MED'INN'PHARMA, which develops a pro-resolutive drug candidate called SuperMapo^®^. FB, SP, and PS are shareholder of MED'INN'PHARMA. The remaining authors declare that the research was conducted in the absence of any commercial or financial relationships that could be construed as a potential conflict of interest.

## References

[1] Smolen JS Aletaha D Mcinnes IB. Rheumatoid arthritis. Lancet. (2016) 388:2023–38. 10.1016/S0140-6736(16)30173-8 27156434

[2] Castaneda S Nurmohamed MT Gonzalez-Gay MA. Cardiovascular disease in inflammatory rheumatic diseases. Best Pract Res Clin Rheumatol. (2016) 30:851–69. 10.1016/j.berh.2016.10.006 27964792

[3] Aletaha D Neogi T Silman AJ Funovits J Felson DT Bingham CO. 2010 Rheumatoid arthritis classification criteria: an American College of Rheumatology/European League Against Rheumatism collaborative initiative. Arthritis Rheum. (2010) 62:2569–81. 10.1002/art.27584 20872595

[4] Smolen JS Breedveld FC Burmester GR Bykerk V Dougados M Emery P. Treating rheumatoid arthritis to target: 2014 update of the recommendations of an international task force. Ann Rheum Dis. (2016) 75:3–15. 10.1136/annrheumdis-2015-207524 25969430 PMC4717393

[5] Smolen JS Van Der Heijde D Machold KP Aletaha D Landewe R. Proposal for a new nomenclature of disease-modifying antirheumatic drugs. Ann Rheum Dis. (2014) 73:3–5. 10.1136/annrheumdis-2013-204317 24072562

[6] Smolen JS Landewe RBM Bijlsma JWJ Burmester GR Dougados M Kerschbaumer A. EULAR recommendations for the management of rheumatoid arthritis with synthetic and biological disease-modifying antirheumatic drugs: 2019 update. Ann Rheum Dis. (2020) 79:685–99. 10.1136/annrheumdis-2019-216655 31969328

[7] Radner H Smolen JS Aletaha D. Remission in rheumatoid arthritis: benefit over low disease activity in patient-reported outcomes and costs. Arthritis Res Ther. (2014) 16:R56. 10.1186/ar4491 24555808 PMC3979137

[8] Listing J Strangfeld A Kary S Rau R Von Hinueber U Stoyanova-Scholz M. Infections in patients with rheumatoid arthritis treated with biologic agents. Arthritis Rheum. (2005) 52:3403–3412. 10.1002/art.21386 16255017

[9] Liu R Zhao P Tan W Zhang M. Cell therapies for refractory rheumatoid arthritis. Clin Exp Rheumatol. (2018) 36:911–9. 29745893

[10] Saas P Kaminski S Perruche S. Prospects of apoptotic cell-based therapies for transplantation and inflammatory diseases. Immunotherapy. (2013) 5:1055–73. 10.2217/imt.13.103 24088076

[11] Saas P Daguindau E Perruche S. Concise review: apoptotic cell-based therapies-rationale, preclinical results and future clinical developments. Stem Cells. (2016) 34:1464–73. 10.1002/stem.2361 27018198

[12] Mcinnes IB Schett G. The pathogenesis of rheumatoid arthritis. N Engl J Med. (2011) 365:2205–19. 10.1056/NEJMra1004965 22150039

[13] Croft AP Campos J Jansen K Turner JD Marshall J Attar M. Distinct fibroblast subsets drive inflammation and damage in arthritis. Nature. (2019) 570:246–51. 10.1038/s41586-019-1263-7 31142839 PMC6690841

[14] Szekanecz Z Pakozdi A Szentpetery A Besenyei T Koch AE. Chemokines and angiogenesis in rheumatoid arthritis. Front Biosci. (Elite Ed). (2009) 1:44–51. 19482623 10.2741/e5 PMC2884394

[15] Mcinnes IB Leung BP Liew FY. Cell-cell interactions in synovitis. Interactions between T lymphocytes and synovial cells. Arthritis Res. (2000) 2:374–8. 10.1186/ar128 11094451 PMC130139

[16] De Rycke L Peene I Hoffman IE Kruithof E Union A Meheus L. Rheumatoid factor and anticitrullinated protein antibodies in rheumatoid arthritis: diagnostic value, associations with radiological progression rate, extra-articular manifestations. Ann Rheum Dis. (2004) 63:1587–93. 10.1136/ard.2003.017574 15547083 PMC1754859

[17] Wegner N Wait R Sroka A Eick S Nguyen KA Lundberg K. Peptidylarginine deiminase from Porphyromonas gingivalis citrullinates human fibrinogen and alpha-enolase: implications for autoimmunity in rheumatoid arthritis. Arthritis Rheum. (2010) 62:2662–72. 10.1002/art.27552 20506214 PMC2941529

[18] Shi J Van Veelen PA Mahler M Janssen GM Drijfhout JW Huizinga TW. Carbamylation and antibodies against carbamylated proteins in autoimmunity and other pathologies. Autoimmun Rev. (2014) 13:225–30. 10.1016/j.autrev.2013.10.008 24176675

[19] Gaujoux-Viala C Nam J Ramiro S Landewe R Buch MH Smolen JS. Efficacy of conventional synthetic disease-modifying antirheumatic drugs, glucocorticoids and tofacitinib: a systematic literature review informing the 2013 update of the EULAR recommendations for management of rheumatoid arthritis. Ann Rheum Dis. (2014) 73:510–5. 10.1136/annrheumdis-2013-204588 24395555 PMC3932966

[20] Nam JL Ramiro S Gaujoux-Viala C Takase K Leon-Garcia M Emery P. Efficacy of biological disease-modifying antirheumatic drugs: a systematic literature review informing the 2013 update of the EULAR recommendations for the management of rheumatoid arthritis. Ann Rheum Dis. (2014) 73:516–28. 10.1136/annrheumdis-2013-204577 24399231

[21] Kerschbaumer A Sepriano A Smolen JS Van Der Heijde D Dougados M Van Vollenhoven R. Efficacy of pharmacological treatment in rheumatoid arthritis: a systematic literature research informing the 2019 update of the EULAR recommendations for management of rheumatoid arthritis. Ann Rheum Dis. (2020) 79:744–59. 10.1136/annrheumdis-2019-216656 32033937 PMC7286044

[22] O'shea JJ Kontzias A Yamaoka K Tanaka Y Laurence A. Janus kinase inhibitors in autoimmune diseases. Ann Rheum Dis. (2013) 72(Suppl. 2): ii111–5. 10.1136/annrheumdis-2012-202576 23532440 PMC3616338

[23] Wang L Wang L Cong X Liu G Zhou J Bai B. Human umbilical cord mesenchymal stem cell therapy for patients with active rheumatoid arthritis: safety and efficacy. Stem Cells Dev. (2013) 22:3192–202. 10.1089/scd.2013.0023 23941289

[24] Alvaro-Gracia JM Jover JA Garcia-Vicuna R Carreno L Alonso A Marsal S. Intravenous administration of expanded allogeneic adipose-derived mesenchymal stem cells in refractory rheumatoid arthritis. (Cx611): results of a multicentre, dose escalation, randomised, single-blind, placebo-controlled phase Ib/IIa clinical trial. Ann Rheum Dis. (2017) 76:196–202. 10.1136/annrheumdis-2015-208918 27269294

[25] Bell GM Anderson AE Diboll J Reece R Eltherington O Harry RA. Autologous tolerogenic dendritic cells for rheumatoid and inflammatory arthritis. Ann Rheum Dis. (2017) 76:227–34. 10.1136/annrheumdis-2015-208456 27117700 PMC5264217

[26] Doran AC Yurdagul A Jr Tabas I. Efferocytosis in health and disease. Nat Rev Immunol. (2020) 20:254–67. 10.1038/s41577-019-0240-6 31822793 PMC7667664

[27] Kourtzelis I Hajishengallis G Chavakis T. Phagocytosis of apoptotic cells in resolution of inflammation. Front Immunol. (2020) 11:553. 10.3389/fimmu.2020.00553 32296442 PMC7137555

[28] Abdolmaleki F Farahani N Gheibi Hayat SM Pirro M Bianconi V Barreto GE. The role of efferocytosis in autoimmune diseases. Front Immunol. (2018) 9:1645. 10.3389/fimmu.2018.01645 30083153 PMC6064952

[29] Lu Q Lemke G. Homeostatic regulation of the immune system by receptor tyrosine kinases of the Tyro 3 family. Science. (2001) 293:306–11. 10.1126/science.1061663 11452127

[30] Ariel A Fredman G Sun YP Kantarci A Van Dyke TE Luster AD. Apoptotic neutrophils and T cells sequester chemokines during immune response resolution through modulation of CCR5 expression. Nat Immunol. (2006) 7:1209–16. 10.1038/ni1392 17013391 PMC1797066

[31] Chen W Frank ME Jin W Wahl SM. TGF-beta released by apoptotic T cells contributes to an immunosuppressive milieu. Immunity. (2001) 14:715–25. 10.1016/S1074-7613(01)00147-9 11420042

[32] Pupjalis D Goetsch J Kottas DJ Gerke V Rescher U. Annexin A1 released from apoptotic cells acts through formyl peptide receptors to dampen inflammatory monocyte activation via JAK/STAT/SOCS signalling. EMBO Mol Med. (2011) 3:102–14. 10.1002/emmm.201000113 21254404 PMC3377061

[33] Krispin A Bledi Y Atallah M Trahtemberg U Verbovetski I Nahari E. Apoptotic cell thrombospondin-1 and heparin-binding domain lead to dendritic-cell phagocytic and tolerizing states. Blood. (2006) 108:3580–9. 10.1182/blood-2006-03-013334 16882710

[34] Medina CB Mehrotra P Arandjelovic S Perry JSA Guo Y Morioka S. Metabolites released from apoptotic cells act as tissue messengers. Nature. (2020) 580:130–5. 10.1038/s41586-020-2121-3 32238926 PMC7217709

[35] Yamaguchi H Maruyama T Urade Y Nagata S. Immunosuppression via adenosine receptor activation by adenosine monophosphate released from apoptotic cells. Elife. (2014) 3:e02172. 10.7554/eLife.02172 24668173 PMC3963506

[36] Han CZ Juncadella IJ Kinchen JM Buckley MW Klibanov AL Dryden K. Macrophages redirect phagocytosis by non-professional phagocytes and influence inflammation. Nature. (2016) 539:570–4. 10.1038/nature20141 27820945 PMC5799085

[37] Lauber K Bohn E Krober SM Xiao YJ Blumenthal SG Lindemann RK. Apoptotic cells induce migration of phagocytes via caspase-3-mediated release of a lipid attraction signal. Cell. (2003) 113:717–30. 10.1016/S0092-8674(03)00422-7 12809603

[38] Truman LA Ford CA Pasikowska M Pound JD Wilkinson SJ Dumitriu IE. CX3CL1/fractalkine is released from apoptotic lymphocytes to stimulate macrophage chemotaxis. Blood. (2008) 112:5026–36. 10.1182/blood-2008-06-162404 18799722

[39] Chekeni FB Elliott MR Sandilos JK Walk SF Kinchen JM Lazarowski ER. Pannexin 1 channels mediate 'find-me' signal release and membrane permeability during apoptosis. Nature. (2010) 467:863–7. 10.1038/nature09413 20944749 PMC3006164

[40] Poon IK Lucas CD Rossi AG Ravichandran KS. Apoptotic cell clearance: basic biology and therapeutic potential. Nat Rev Immunol. (2014) 14:166–80. 10.1038/nri3607 24481336 PMC4040260

[41] Huynh ML Fadok VA Henson PM. Phosphatidylserine-dependent ingestion of apoptotic cells promotes TGF-beta1 secretion and the resolution of inflammation. J Clin Invest. (2002) 109:41–50. 10.1172/JCI0211638 11781349 PMC150814

[42] Voll RE Herrmann M Roth EA Stach C Kalden JR Girkontaite I. Immunosuppressive effects of apoptotic cells. Nature. (1997) 390:350–1. 10.1038/37022 9389474

[43] Fadok VA Bratton DL Konowal A Freed PW Westcott JY Henson PM. Macrophages that have ingested apoptotic cells *in vitro* inhibit proinflammatory cytokine production through autocrine/paracrine mechanisms involving TGF-beta, PGE2, and PAF. J Clin Invest. (1998) 101:890–8. 10.1172/JCI1112 9466984 PMC508637

[44] Craciun LI Digiambattista M Schandene L Laub R Goldman M Dupont E. Anti-inflammatory effects of UV-irradiated lymphocytes: induction of IL-1Ra upon phagocytosis by monocyte/macrophages. Clin Immunol. (2005) 114:320–6. 10.1016/j.clim.2004.11.006 15721843

[45] Lee YJ Moon C Lee SH Park HJ Seoh JY Cho MS. Apoptotic cell instillation after bleomycin attenuates lung injury through hepatocyte growth factor induction. Eur Respir J. (2012) 40:424–35. 10.1183/09031936.00096711 22441736

[46] Saas P Bonnefoy F Toussirot E Perruche S. Harnessing apoptotic cell clearance to treat autoimmune arthritis. Front Immunol. (2017) 8:1191. 10.3389/fimmu.2017.01191 29062314 PMC5640883

[47] Gray M Miles K Salter D Gray D Savill J. Apoptotic cells protect mice from autoimmune inflammation by the induction of regulatory B cells. Proc Natl Acad Sci U S A. (2007) 104:14080–5. 10.1073/pnas.0700326104 17715067 PMC1955797

[48] Perruche S Saas P Chen W. Apoptotic cell-mediated suppression of streptococcal cell wall-induced arthritis is associated with alteration of macrophage function and local regulatory T-cell increase: a potential cell-based therapy? Arthritis Res Ther. (2009) 11:R104. 10.1186/ar2750 19570235 PMC2745779

[49] Notley CA Brown MA Wright GP Ehrenstein MR. Natural IgM is required for suppression of inflammatory arthritis by apoptotic cells. J Immunol. (2011) 186:4967–72. 10.4049/jimmunol.1003021 21383247

[50] Notley CA Brown MA Mcgovern JL Jordan CK Ehrenstein MR. Engulfment of activated apoptotic cells abolishes TGF-beta-mediated immunoregulation via the induction of IL-6. J Immunol. (2015) 194:1621–7. 10.4049/jimmunol.1401256 25601923 PMC4319310

[51] Bonnefoy F Daoui A Valmary-Degano S Toussirot E Saas P Perruche S. Apoptotic cell infusion treats ongoing collagen-induced arthritis, even in the presence of methotrexate, and is synergic with anti-TNF therapy. Arthritis Res Ther. (2016) 18:184. 10.1186/s13075-016-1084-0 27516061 PMC4982016

[52] Hilliard KA Brown CR. Treatment of Borrelia burgdorferi-infected mice with apoptotic cells attenuates lyme arthritis via PPAR-gamma. J Immunol. (2019) 202:1798–806. 10.4049/jimmunol.1801179 30700583

[53] Wu Z Ma HM Kukita T Nakanishi Y Nakanishi H. Phosphatidylserine-containing liposomes inhibit the differentiation of osteoclasts and trabecular bone loss. J Immunol. (2010) 184:3191–201. 10.4049/jimmunol.0803609 20176740

[54] Ma HM Wu Z Nakanishi H. Phosphatidylserine-containing liposomes suppress inflammatory bone loss by ameliorating the cytokine imbalance provoked by infiltrated macrophages. Lab Invest. (2011) 91:921–31. 10.1038/labinvest.2011.54 21464820

[55] Bonnefoy F Gauthier T Vallion R Martin-Rodriguez O Missey A Daoui A. Factors produced by macrophages eliminating apoptotic cells demonstrate pro-resolutive properties and terminate ongoing inflammation. Front Immunol. (2018) 9:2586. 10.3389/fimmu.2018.02586 30542342 PMC6277856

[56] Bonnefoy F Perruche S Couturier M Sedrati A Sun Y Tiberghien P. Plasmacytoid dendritic cells play a major role in apoptotic leukocyte-induced immune modulation. J Immunol. (2011) 186:5696–705. 10.4049/jimmunol.1001523 21460208

[57] Saas P Chague C Maraux M Cherrier T. Towards the characterization of human pro-resolving macrophages? Front Immunol. (2020) 11:593300. 10.3389/fimmu.2020.593300 33281821 PMC7691375

[58] Mevorach D Zuckerman T Reiner I Shimoni A Samuel S Nagler A. Single infusion of donor mononuclear early apoptotic cells as prophylaxis for graft-versus-host disease in myeloablative HLA-matched allogeneic bone marrow transplantation: a phase I/IIa clinical trial. Biol Blood Marrow Transplant. (2014) 20:58–65. 10.1016/j.bbmt.2013.10.010 24140121

